# The Association Between Documentation of Koplik Spots and Laboratory Diagnosis of Measles and Other Rash Diseases in a National Measles Surveillance Program in Japan

**DOI:** 10.3389/fmicb.2019.00269

**Published:** 2019-02-18

**Authors:** Hirokazu Kimura, Komei Shirabe, Makoto Takeda, Miho Kobayashi, Hiroyuki Tsukagoshi, Kaori Okayama, Akihide Ryo, Koo Nagasawa, Nobuhiko Okabe, Hiroko Minagawa, Kunihisa Kozawa

**Affiliations:** ^1^Infectious Disease Surveillance Center, National Institute of Infectious Diseases, Musashimurayama, Japan; ^2^Graduate School of Health Science, Gunma Paz University, Takasaki, Japan; ^3^Department of Microbiology, Graduate School of Medicine, Yokohama City University, Yokohama, Japan; ^4^Yamaguchi Prefectural Institute of Public Health and Environment, Yamaguchi, Japan; ^5^Department of Virology III, National Institute of Infectious Diseases, Musashimurayama, Japan; ^6^Gunma Prefectural Institute of Public Health and Environmental Sciences, Maebashi, Japan; ^7^Eastern Chiba Medical Center, Togane, Japan; ^8^Kawasaki City Institute for Public Health, Kawasaki, Japan; ^9^Aichi Prefectural Institute of Public Health, Nagoya, Japan

**Keywords:** measles, Koplik spots, RT-PCR, rash and fever, viral infection

## Abstract

Koplik spots are considered a disease-specific sign for measles, although comprehensive virological studies have not been conducted to date. In Japan, a national survey of 3023 measles and measles-suspected cases was conducted between 2009 and 2014 using polymerase chain reaction (PCR) or reverse transcription PCR (RT-PCR) to detect various rash/fever-associated viruses. Koplik spots were observed in 717 of 3023 cases (23.7%). Among these, the measles virus was detected in 202 cases (28.2%), while the rubella virus was detected in 125 cases (17.4%). Other viruses were detected in 51 cases having the spots (7.1%). In some of the cases with spots, two or three viruses, such as the rubella virus, parvovirus, and human herpesvirus type 6 were also detected. The sensitivity and specificity of Koplik spots as a diagnostic marker for measles were 48 and 80%, respectively. The results suggested that Koplik spots might appear not only in measles but also in other viral infections, such as rubella, as a clinical sign.

## Introduction

Measles is a viral disease characterized by systemic rash, high fever, and respiratory and conjunctival symptoms (Griffin, 2013). Among physical signs, Koplik spots on the buccal mucous membrane could be accepted by pediatricians and physicians as a disease-specific sign “pathognomonic” for measles (Mason, 2011). Koplik spot was introduced by Henry E. Koplik in 1896 (Markel, 2015). Bluish-white specks on the buccal mucosa near the molars appear a few days before the appearance of the distinctive rash of measles. Therefore, the sign is extremely useful for the early diagnosis of measles (Xavier and Forgie, 2015). However, some studies suggested that the sign might not be disease-specific (Annunziato, 1987; Evans et al., 1992). For example, Evans et al. (1992) reported a case of Koplik spots associated with parvovirus B19 infection. However, these reports might have a limitation, such as patient numbers.

Recent genetic technologies, including polymerase chain reaction method (PCR), enabled the detection of various viruses with specific and high sensitivity. Using these methods, we analyzed the correlation between Koplik spots and detected viruses from over 3000 cases of measles-suspected patients based on the 6-year national study in Japan.

## Materials and Methods

### Sample Collections

From January 2009 to September 2014, the national surveillance data of 5604 cases of measles, including clinically diagnosed or PCR-confirmed, were provided by 74 public health institutes of local governments around Japan. The clinically diagnosed measles-suspected cases were to be reported immediately to local governments on a prescribed official form of a national surveillance scheme, with a set of specimens of the nasopharyngeal swab, whole blood, and urine, forwarded to the local public health institutes for PCR analysis. Of 5604 cases, we excluded patients with unclear records of the disease onset or symptoms, whose specimens were obtained 8 days after the disease onset, and those who only provided one specimen from the swab, serum, or urine samples for PCR confirmation. Consequently, we enrolled 3023 cases for the analysis in this study.

Thus, at least, two samples of swab, blood, and urine were obtained from all patients within 7 days of the onset of fever. In addition, we performed a questionnaire survey of clinical signs and disease courses to reassess the diagnosis. The questionnaire collected information about age, sex, clinical symptoms, date of the disease onset, sampling date, specimen type used for PCR, and co-detected pathogens other than the measles virus.

### Virus Detection

The measles virus (MeV) *N* gene detection was performed using reverse transcription-PCR (RT-PCR) as described previously (Morita et al., 2007; Taira et al., 2008). Briefly, viral genomes were extracted using an available kit (QIAamp Viral RNA Mini Kit; Qiagen, Valencia, CA) and subjected to RT-PCR test. The primer sets for the first and nested PCR were as described previously (Morita et al., 2007). PCR was performed under the following conditions: 30 cycles at 98°C for 10 s, 53°C for 30 s, 72°C for 1 min. Moreover, to avoid cross-contamination of the MeV genome, we conducted every test using the negative control (no viral genome) and positive control for the artificial synthetic RNA inserted *Bam*HI digestion sequences. Besides, we attempted further detection of other viruses that could cause rash and fever in measles-negative cases, including the rubella virus (RuV), human herpesvirus 6 and 7, and human parvovirus B19, using PCR or RT-PCR (Koch and Adler, 1990; Okuno et al., 1995; Tanaka-Taya et al., 1996; McIver et al., 2005; World Health Organization, 2007; Yasui et al., 2014). The primers are shown in Supplementary Table S1.

### Ethics Statement

To diagnose measles, all samples were primarily collected under compliance with Act on the Prevention of Infectious Diseases and Medical Care for Patients with Infectious Diseases of Japan. Informed consent was obtained from all participants, which was obtained from the subjects or their legally acceptable representatives for sample donation. The patients’ data were anonymized. To perform extraneous study (this study) and due to the lack of written informed consent, the present study protocols were deliberated by the Ethics Committee on Human Research of the Gunma Prefectural Institute of Public Health (Gunma, Japan). Finally, this study had no infringement of the patient’s rights and was approved by the research ethics committee (approved no. H26-30056-18). In addition, all methods were performed in accordance with the approved guidelines.

## Results

### Demographic Data of Cases

Supplementary Table S2 summarizes the demographic data of the study cohort. All patients invariably had both fever and rash, whereas Koplik spots were reported in approximately 24% (717 cases) of all cases. The vaccination history for measles was unclear in most cases.

### Virus Detection and Koplik Spots

Measles virus was detected in nearly 28% (202 cases) of cases that reported Koplik spots (Table 1). RuV was detected in about 17% (125 cases) of patients displaying the spots. In addition, other viruses, such as parvovirus B19 and human herpesvirus 6/7, were detected in approximately 7% (51 cases). Moreover, Table 1 presents viruses detected in cases that exhibited Koplik spots, but MeV was negative in PCR tests. We observed the codetection of RuV, parvovirus B19, human herpesvirus 6/7, and RS virus in several cases. In this study, the sensitivity and specificity of Koplik spots were 48.0 and 80.2%, respectively (Supplementary Table S3). Furthermore, the positive rates of Koplik spots in patients with other viruses detected were approximately 20–30% (Table 2). These findings suggested that Koplik spots were not a specific manifestation of measles.

**Table 1:** Viruses detected in patients with Koplik spots.

Detected viruses	No. of cases
Measles virus	202
Rubella virus	125
Rubella virus + Parvovirus B19	2
Rubella virus + Herpesvirus 6	1
Rubella virus + Herpesvirus 7	1
Parvovirus B19	14
Parvovirus B19 + Herpesvirus 6 + Herpesvirus 7	1
Parvovirus B19 +RS virus	1
Herpesvirus 6	9
Herpesvirus 7	7
Coxsackievirus group A	6
Adenovirus	4
Rhinovirus	3
RS virus	2
Herpes simplex virus	1
Varicella Zoster virus	1
Parainfluenza virus	1
Influenza virus	1
Enterovirus	1
Cytomegalovirus	1
Total	378


**Table 2:** Association between Koplik spots and detected viruses.

	Koplik spots		Total	Positive rate^∗^ (%)	Percentage of patients with Koplik spots (%)
	Positive	Negative			
Measles virus	202	219	421	48.0	28.2
Rubella virus	125	474	599	20.9	17.4
Parvovirus B19	16	34	50	32.0	2.2
Others	41	177	218	18.8	5.7
Total	384	904	1,288		


*^∗^Positive rate = number of patients with Koplik signs/number of patients with positive PCR results.*

## Discussion

This study is based on the national data of Japan obtained through the country’s efforts toward the elimination of measles, which started in 2009. The annual estimation of measles cases reported >10,000 cases in Japan until 2008 (Centers for Disease Control and Prevention [CDC], 2008). In 2007, a major measles outbreak, which was primarily reported in individuals in their teens and twenties, led the Japanese government to set goals for the elimination of measles and act to reinforce its vaccination policy (Morita et al., 2007; Centers for Disease Control and Prevention [CDC], 2008). In addition, the surveillance policy was reinforced from a sentinel survey system to an all-case survey system in 2008. Going forward, laboratory confirmation by using PCR tests was introduced in 2008 and extended to all suspected cases of measles in 2010. Consequently, the outcome was remarkable. Finally, the WHO approved the elimination of the disease in March, 2015 in Japan.

Until the 1970s or 1980s, measles was primarily diagnosed according to physical signs and clinical courses and was occasionally confirmed by epidemiological links. Typically, after 2–3 days since the presentation of respiratory symptoms and fever, the maculopapular rash appears in the face and spreads to the trunk and extremities. This rash is a distinct characteristic of measles, and it is a useful finding for diagnosis. However, patients with measles who received vaccine often do not present with the typical rash (Leung et al., 2018). The diagnosis of measles can be challenging in areas with low incidence and high vaccine coverage (Leung et al., 2018). Thus, at present, the RT-PCR method provides direct and indisputable detection of the measles virus in human specimens. A set of PCR tests of serum, urine, and throat swab, if obtained timely and adequately, from the onset of fever until day 7 of the disease, provides concrete proof of the infection. If all PCR results are negative, measles can be excluded with utmost certainty. In addition, we investigated other viruses in MeV-negative cases. Through these further investigations, RuV, herpesvirus, parvovirus, and some other viruses that could cause rash and fever syndrome were detected (Table 1), demonstrating that Koplik spots could appear not only in measles but also in other viral infection, such as rubella, although the specificity of this clinical sign was relatively high (around 80%).

Koplik spot that is associated with measles virus infection may be attributed to the destruction of glandular epithelial cells and phlebectasia around the submucosal gland duct (Xavier and Forgie, 2015). In contrast, eruption due to rubella virus infection is caused by superficial perivascular dermatitis (Won et al., 2001). Pathologically, perivascular inflammation based on lymphocytes is consistent with measles. Therefore, the pathophysiology of rubella and measles may be similar. Based on these findings, Koplik spots may also be observed in individuals with rubella infection, although studies about the pathological findings of enanthema due to rubella have not been conducted (Cherry, 2004).

Next, we could not obtain photographs of Koplik spots. Therefore, whether there were differences in Koplik spots due to viruses was not validated. To know more information about this, further studies must be conducted.

For experienced physicians and pediatricians, in particular, diagnosing a typical measles case is relatively easy and precise even without laboratory confirmation. However, physicians with the treatment experiences of measles may decrease in the many countries which were approved elimination of the disease including Japan. Meanwhile, modified measles may increase near future because vaccine coverage of measles has been increased. Thus, it may become more difficult to diagnose measles by clinical findings alone such as Koplik spots. In conclusion, this study suggests that the diagnosis of measles or other viral infections should be based on not only the collective information of clinical manifestations but also laboratory confirmation.

## Author Contributions

HK and KK designed the study. KS, MT, MK, HT, KO, KN, NO, and HM analyzed the data. MK, HT, and HK contributed analytic tools. HK, KK, and AR wrote the manuscript.

## Conflict of Interest Statement

The authors declare that the research was conducted in the absence of any commercial or financial relationships that could be construed as a potential conflict of interest.
