# Supplementary material for: The Association Between Documentation of Koplik Spots and Laboratory Diagnosis of Measles and Other Rash Diseases in a National Measles Surveillance Program in Japan
Source: Front Microbiol. 2019 Feb 18;10:269. doi: 10.3389/fmicb.2019.00269 (PMC6387930; doi:10.3389/fmicb.2019.00269)
Supplement: Supplementary file 1 [file Table_1.docx]

Supplementary Material

The association between documentation of Koplik's spots and laboratory diagnosis of measles and other rash diseases in a national measles surveillance program in Japan

**Hirokazu Kimura, Komei Shirabe, Makoto Takeda, Miho Kobayashi, Hiroyuki Tsukagoshi, Kaori Okayama, Akihide Ryo, Koo Nagasawa, Nobuhiko Okabe, Hiroko Minagawa, and Kunihisa Kozawa***

***Correspondence:** Kunihisa Kozawa: kkozawa-gi@umin.net

# Supplementary Tables

## Supplementary Table 1

## Table S1. Primers used in this study.

| virus | Primer | | Sequence (5'→3') |
| --- | --- | --- | --- |
| Rubella virus | 1st PCR　Forward primer | NSL F3 | TCC TTG CGC CGA AGA CT |
|  | 1st PCR　Reverse primer | NSL B3-6 | AGA GGG GGT CCA CTT GAG |
|  | Nested PCR Forward primer | F2 nest | CCA CTG AGA CCG GCT GCG A |
|  | Nested PCR Reverse primer | B2 nest | GCC TCG GGG AGG AAG ATG AC |
| Human herpesvirus 6 | 1st PCR　Forward primer | 6A | TTCTCCAGATGTGCCAGGGAAATCC |
|  | 1st PCR　Reverse primer | 6C | CATCATTGTTATCGC TTTCACTCTC |
|  | Nested PCR Forward primer | 6B | AGTGACAGATCTGGGCGGCCCTAATAACTT |
|  | Nested PCR Reverse primer | 6E | AGGTGCTGAGTGATCAGTTTCATAACCAAA |
| Human herpesvirus 7 | 1st PCR　Forward primer | 7F3 | AGTTCCAGCACTGCAATCG |
|  | 1st PCR　Reverse primer | 7R3 | CACAAAAGCGTCGCTATCAA |
|  | Nested PCR Forward primer | 7F4 | CGCATACACCAACCCTACTG |
|  | Nested PCR Reverse primer | 7R4 | GACTCATTATGGGGATCGAC |
| Human herpesvirus 8 | 1st PCR　Forward primer | HHV8EF | CCAGCTAGCAGTGCTACCCCCATT |
|  | 1st PCR　Reverse primer | HHV8ER | ATGGACAGATCGTCAAGCACTCGC |
|  | Nested PCR Forward primer | KS1 | AGCCGAAAGGATTCCACCAT |
|  | Nested PCR Reverse primer | KS2 | TCCGTGTTGTCTAGTCCAG |
| Parvovirus B19 | Forward primer | K-1 | ATAAATCCATATACTCATT |
|  | Reverse primer | K-2 | CTAAAGTATCCTGACCTTG |

## Supplementary Table 2

**Table S2**. Demographic data of measles-suspected cases in this study

| Reported year | No. of cases | Age  (Mean ± SD) | Sex  (M/F) | No. of cases with  Koplik spots | | Measles confirmed  by PCR (%) | |
| --- | --- | --- | --- | --- | --- | --- | --- |
| 2009 | 33 | 13.9 ± 17.0 | 18/15 | 4 | (12.1%) | 0 | (0.0%) |
| 2010 | 132 | 13.9 ± 15.2 | 67/65 | 22 | (16.7%) | 0 | (0.0%) |
| 2011 | 466 | 18.5 ± 16.8 | 272/194 | 120 | (25.8%) | 5 | (1.1%) |
| 2012 | 635 | 18.6 ± 16.0 | 375/260 | 148 | (23.3%) | 23 | (3.6%) |
| 2013 | 839 | 25.4 ± 17.3 | 571/268 | 168 | (20.0%) | 46 | (5.5%) |
| 2014 | 918 | 15.2 ± 15.9 | 459/459 | 255 | (27.8%) | 347 | (37.8%) |
| Total | 3,023 | 19.2 ± 16.9 | 1,762/1261 | 717 | (23.7%) | 421 | (13.9%) |

**1.3 Supplementary Table3**

**Table S3**. The sensitivity and specificity of Koplik spots for measles

|  | Measles virus detected | | Total |
| --- | --- | --- | --- |
|  | Positive | Negative |  |
| Positive for  Koplik spots | 202 | 515 | 717 |
| Negative for  Koplik spots | 219 | 2,087 | 2,306 |
| Total | 421 | 2,602 | 3,023 |

Sensitivity: 48.0%, Specificity: 80.2%
